# Post-Hoc Analysis of Zuranolone Efficacy and Safety by Baseline MADRS Depressive Symptom Severity in SKYLARK and ROBIN

**DOI:** 10.1192/j.eurpsy.2026.11792

**Published:** 2026-07-31

**Authors:** K. M. Deligiannidis, J. A. Ramos-Quiroga, B. Nair, A. Artemyeva, K. LaGuerre, H. Finnigan, F. Forrestal, J. Jung, V. Nguyen, M. Adams

**Affiliations:** 1 Department of Psychiatry, Zucker Hillside Hospital, Glen Oaks, United States; 2 Department of Psychiatry, Hospital Universitari Vall d’Hebron, Barcelona, Spain; 3 Kent and Medway Mental Health NHS Trust, Maidstone, United Kingdom; 4 Biogen Inc., Cambridge, United States

## Abstract

**Introduction:**

Postpartum depression (PPD) is a common perinatal condition with significant consequences for the patient, child, and family. The efficacy and safety of zuranolone, an oral, once-daily, 14-day treatment for PPD in adults, was assessed in the pivotal SKYLARK and ROBIN studies. The Hamilton Depression Rating Scale 17 (HAMD-17) and Montgomery-Asberg Depression Rating Scale (MADRS) were the primary and a secondary endpoint, respectively. Both studies enrolled participants with a HAMD-17 score ≥ 26 (indicative of severe depressive symptoms). However, the efficacy and safety of zuranolone in patients with moderate PPD remains uncertain.

**Objectives:**

This post-hoc analysis of SKYLARK and ROBIN aimed to characterize the efficacy and safety of zuranolone in patients with PPD with moderate or severe depressive symptoms defined by baseline MADRS score.

**Methods:**

Two severity subgroups were defined based on baseline MADRS total score: moderate (20-34) and severe (≥ 35) depressive symptoms. Changes from baseline (CFB) in HAMD-17 and in MADRS total scores were analyzed in each subgroup using a mixed-effects model for repeated measures. Treatment-emergent adverse events were evaluated by subgroup.

**Results:**

Both SKYLARK and ROBIN showed similar demographic and baseline characteristics in the moderate and severe subgroups, with a balanced distribution of moderate and severe MADRS scores. In each study, the least squares mean (LSM) treatment differences in CFB in HAMD-17 and MADRS total scores for each subgroup favored zuranolone at Day 15, with effects sustained to Day 45 (Tables 1 and 2). No clinically meaningful differences in safety were observed across the subgroups in either study.

**Tables 1 and 2.** LSM Treatment Differences in CFB versus placebo in HAMD-17 and MADRS Total Scores at Days 15 and 45 Tables 1 and 2. Long description.

SKYLARK	HAMD-17	p-value	MADRS	p-value
**Day 15**				
**Moderate**	-4.4 (n=87)	0.005	-5.2 (n=87)	0.02
**Severe**	-4.0 (n=95)	0.03	-5.0 (n=94)	0.06
**Day 45**				
**Moderate**	-3.8 (n=80)	0.03	-4.5 (n=80)	0.05
**Severe**	-3.4 (n=88)	0.08	-5.0 (n=88)	0.08

ROBIN	HAMD-17	p-value	MADRS	p-value
**Day 15**				
**Moderate**	-3.2 (n=60)	0.09	-3.1 (n=60)	0.20
**Severe**	-4.6 (n=87)	0.02	-5.7 (n=87)	0.04
**Day 45**				
**Moderate**	-3.6 (n=58)	0.06	-4.6 (n=58)	0.06
**Severe**	-4.3 (n=84)	0.02	-7.1 (n=84)	0.01

**Conclusions:**

Post hoc analyses suggested that the clinical effects of zuranolone treatment versus placebo could be applied to patients with moderate or severe depressive symptoms.

**Disclosure of Interest:**

K. Deligiannidis Consultant of: GH Research, Sage Therapeutics, Inc., Brii Biosciences, Inc., Lipocine, Gerbera Therapeutics, Reunion Neuroscience, Neurocentria, Brainify, and NextSense, J. A. Ramos-Quiroga Grant / Research support from: Department of Psychiatry chaired by him received unrestricted educational and research support from the following companies in the last 3 years: Exeltis, Idorsia, Casen-Recordati, Takeda, Neuraxpharm, Oryzon, Roche, Probitas, Rubió, and Johnson & Johnson., Consultant of: Biogen, Idorsia, Casen-Recordati, Johnson & Johnson, Novartis, Takeda, Bial, Sincrolab, Neuraxpharm, Novartis, Lilly, BMS, Medice, Rubió, Uriach, Technofarma, and Raffo, Speakers bureau of: Biogen, Idorsia, Casen-Recordati, Johnson & Johnson, Novartis, Takeda, Bial, Sincrolab, Neuraxpharm, Novartis, Lilly, BMS, Medice, Rubió, Uriach, Technofarma, and Raffo, B. Nair: None Declared, A. Artemyeva Employee of: Biogen Inc. and may hold stock, K. LaGuerre Employee of: Biogen Inc. and may hold stock, H. Finnigan Employee of: Biogen Inc. and may hold stock, F. Forrestal Employee of: Biogen Inc. and may hold stock, J. Jung Employee of: Biogen Inc. and may hold stock, V. Nguyen Employee of: Biogen Inc. and may hold stock, M. Adams Employee of: Biogen Inc. and may hold stock

## Long descriptions

### Tables 1 and 2. Long description

The first table, SKYLARK, shows results for Day 15 and Day 45. At Day 15, Moderate cases (n=87) had an H A M D 17 difference of minus 4.4 (p=0.005) and a M A D R S difference of minus 5.2 (p=0.02). Severe cases (n=95) had an H A M D 17 difference of minus 4.0 (p=0.03) and a M A D R S difference of minus 5.0 (p=0.06). At Day 45, Moderate cases (n=80) showed minus 3.8 (p=0.03) for H A M D 17 and minus 4.5 (p=0.05) for M A D R S. Severe cases (n=88) showed minus 3.4 (p=0.08) for H A M D 17 and minus 5.0 (p=0.08) for M A D R S.

The second table, ROBIN, shows results for Day 15 and Day 45. At Day 15, Moderate cases (n=60) had an H A M D 17 difference of minus 3.2 (p=0.09) and a M A D R S difference of minus 3.1 (p=0.20). Severe cases (n=87) had an H A M D 17 difference of minus 4.6 (p=0.02) and a M A D R S difference of minus 5.7 (p=0.04). At Day 45, Moderate cases (n=58) showed minus 3.6 (p=0.06) for H A M D 17 and minus 4.6 (p=0.06) for M A D R S. Severe cases (n=84) showed minus 4.3 (p=0.02) for H A M D 17 and minus 7.1 (p=0.01) for M A D R S.

Navigate back to Tables 1 and 2..

